# Brain-enriched guanylate kinase-associated protein, a component of the post-synaptic density protein complexes, contributes to learning and memory

**DOI:** 10.1038/s41598-023-49537-9

**Published:** 2023-12-12

**Authors:** Tayo Katano, Kohtarou Konno, Keizo Takao, Manabu Abe, Akari Yoshikawa, Tsuyoshi Miyakawa, Kenji Sakimura, Masahiko Watanabe, Seiji Ito, Takuya Kobayashi

**Affiliations:** 1 Department of Medical Chemistry, Kansai Medical University, Hirakata, Japan; 2 Department of Anatomy, Faculty of Medicine, Hokkaido University, Sapporo, Japan; 3 Section of Behavior Patterns, National Institute of Physiological Sciences, NINS, Okazaki, Japan; 4 Department of Behavioral Physiology, Faculty of Medicine, Life Science Research Center, University of Toyama, Toyama, Japan; 5 Department of Animal Model Development, Brain Research Institute, Niigata University, Niigata, Japan; 6 Division of Systems Medical Science, Institute for Comprehensive Medical Science, Fujita Health University, Toyoake, Japan; 7 Department of Anesthesiology, Osaka Medical and Pharmaceutical University, Takatsuki, Japan

**Keywords:** Learning and memory, Molecular neuroscience, Synaptic plasticity, Neuroscience

## Abstract

Brain-enriched guanylate kinase-associated protein (BEGAIN) is highly enriched in the post-synaptic density (PSD) fraction and was identified in our previous study as a protein associated with neuropathic pain in the spinal dorsal horn. PSD protein complexes containing *N*-methyl-d-aspartate receptors are known to be involved in neuropathic pain. Since these PSD proteins also participate in learning and memory, BEGAIN is also expected to play a crucial role in this behavior. To verify this, we first examined the distribution of BEGAIN in the brain. We found that BEGAIN was widely distributed in the brain and highly expressed in the dendritic regions of the hippocampus. Moreover, we found that BEGAIN was concentrated in the PSD fraction of the hippocampus. Furthermore, immunoelectron microscopy confirmed that BEGAIN was localized at the asymmetric synapses. Behavioral tests were performed using BEGAIN-knockout (KO) mice to determine the contribution of BEGAIN toward learning and memory. Spatial reference memory and reversal learning in the Barns circular maze test along with contextual fear and cued fear memory in the contextual and cued fear conditioning test were significantly impaired in BEGAIN-KO mice compared to with those in wild-type mice. Thus, this study reveals that BEGAIN is a component of the post-synaptic compartment of excitatory synapses involved in learning and memory.

## Introduction

The postsynaptic region of excitatory neurons is an assembly of more than 1000 proteins, including receptors, enzymes, scaffolding proteins, and structural proteins of the cytoskeleton^1–3^. The proteins in this region are arranged as protein–protein interaction complexes, the organization of which is regulated by the action of activity-dependent signaling networks^4,5^. These networks serve as important regulators of synaptic plasticity. We identified brain enriched guanylate kinase-associated protein (BEGAIN) as a neuropathic pain-related protein in the post-synaptic density (PSD) fraction of the spinal dorsal horn^6^. This BEGAIN was first identified by yeast two-hybrid system analysis as a molecule that interacts with the guanylate kinase (GK) domain of PSD-95^7^. We have shown that BEGAIN is highly expressed in the PSD fraction of spinal dorsal horn, and it functionally interacts with *N*-methyl-d-aspartate (NMDA) receptors, including the GluN2B subunit, in the state of neuropathic pain^6^.

Long-term potentiation (LTP), a phenomenon in which synaptic efficacy is enhanced in an activity-dependent manner, has been studied extensively in order to understand synaptic plasticity^8,9^. LTP is a potential component of the cellular mechanism of learning and memory. Inhibition or deletion of relevant proteins in the PSD affects LTP and learning and memory^10–12^. Phosphorylation of the tyrosine residue at 1472 of GluN2B has been reported to increase after LTP in the CA1 and amygdala. On the other hand, fear-dependent memory is impaired by inhibiting phosphorylation at Y1472 of GluN2B in knock-in (GluN2B Y1472F-KI) mice^13,14^. Inhibition of the interaction with α-actinin2, which anchors NMDA receptors (NMDARs) to the synapse, alters the synaptic localization of GluN2B NMDARs. This interaction is attenuated in the GluN2B Y1472F-KI mice. In addition, effective calcium influx and downstream signaling are also reduced in the KI mice^14,15^.

Central sensitization is a plastic change in the somatosensory nervous system in which inflammation or nerve injury enhances synaptic transmission and neuronal circuitry in the nociceptive pathway. Hyperalgesia and allodynia, symptoms of chronic pain, are observed in vivo as a result of central sensitization^16^. Although many of the proteins reported to be involved in hippocampal LTP are also involved in central sensitization of the spinal cord, several molecules, including neurokinin-1, prostaglandin EP, interleukin-1 receptors, and several kinases, function differently in each event^17^. Phosphorylation of Y1472 in GluN2B is increased during neuropathic pain with mechanical allodynia, and inhibition of its phosphorylation significantly suppresses mechanical allodynia^6,15,18,19^. However, the phosphorylated GluN2B downstream signaling in neuropathic pain is not fully understood. Moreover, its role in learning and memory also remains unknown. Therefore, understanding the molecular mechanisms underlying pathophysiological pain is not only important for discernment of neurological disorders such as chronic pain, but also for comprehension of physiological events and memory processes.

Our previous study confirmed that BEGAIN expression was increased in the PSD fraction of the spinal dorsal horn in the neuropathic pain, and this increase was suppressed in Y1472F-KI mice^6^. Since GluN2B-NMDA receptors are involved in learning and memory as well as in chronic pain^20^, we hypothesized that BEGAIN would also participate in learning and memory. On the other hand, its distribution, synaptic localization and role in the brain remain unclear. Therefore, the aim of this study was to confirm the distribution and synaptic localization of BEGAIN by histological analysis. In addition, to clarify the involvement of BEGAIN in learning and memory, we performed behavioral analysis using BEGAIN-knockout (BEGAIN-KO) mice.

## Results

### Generation of BEGAIN-Cre-recombinase driver-Venus reporter mouse

**Figure 1:**
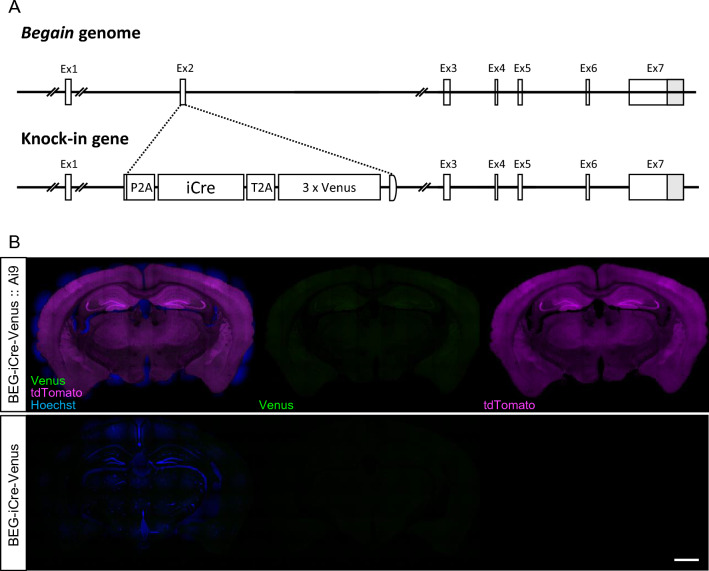
Generation of BEGAIN-iCre-Venus mouse. (**A**) Schematic representation of the structures of brain-enriched guanylate kinase-associated protein (*Begain*) and knock-in genes that are inserted into *improved Cre recombinase* (*iCre*) and three tandem *Venus* genes. (**B**) Histochemistry for BEGAIN-iCre-Venus::Ai9 and BEGAIN-iCre-Venus mice. Expression of iCre is confirmed as tdTomato (upper). Scale bar: 1000 μm.

Previous immunohistochemical analysis could not identify BEGAIN-positive neurons as the BEGAIN protein tended to be localized in the neuronal projections in the spinal cord; however, its localization in the soma was also not detected^6^. To elucidate the BEGAIN-expressing neurons and the distribution of BEGAIN in the central nervous system (CNS), we generated BEGAIN-iCre-Venus mice. In these mice, transcription of improved Cre-recombinase (iCre) and tandemly triplicated Venus (3 × Venus) were driven by the promoter of the BEGAIN gene (Fig. 1A). Unfortunately, BEGAIN-expressing cells could not be visualized by the fluorescent signal of the Venus protein (Fig. 1B lower), suggesting that the amount of Venus expression was not sufficient to visualize BEGAIN-expressing regions in the brain. Therefore, we crossed the driver mice with Ai9 reporter mice to visualize BEGAIN-positive neurons by fluorescence of tdTomato (Fig. 1B upper). TdTomato was detected as a sufficient fluorescent signal for visualization in almost all regions of the coronal section of the brain, suggesting that BEGAIN expression was found to be widely distributed in the brain.

### Distribution of BEGAIN in the hippocampus and the spinal dorsal horn

**Figure 2:**
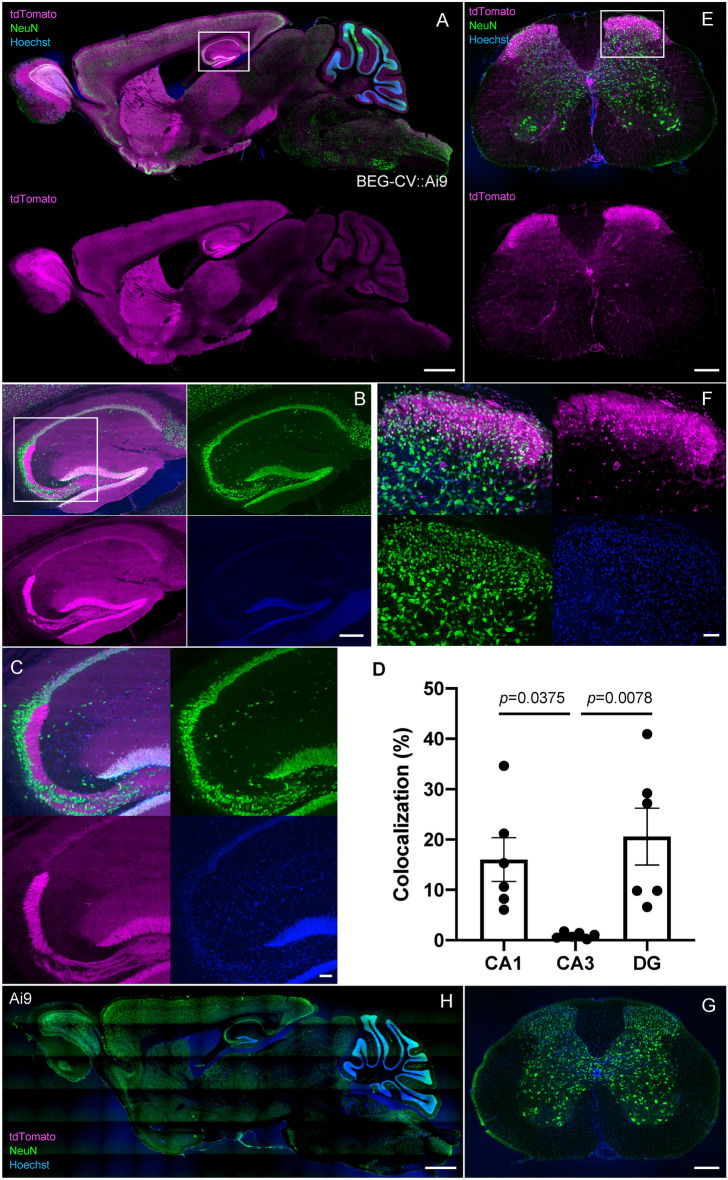
Visualization of the BEGAIN-expressed area using BEGAIN-iCre-driver mouse. Sagittal sections of the brain and transverse sections of the spinal cord were prepared from brain-enriched guanylate kinase-associated protein (BEGAIN)-improved Cre recombinase (iCre)::Ai9 mice. The BEGAIN-expressed area was visualized as the tdTomato-positive area in the brain (**A**–**C**) and the spinal cord (**E**,**F**). Neuronal soma and all cell soma were labeled with the anti-NeuN antibody and Hoechst dye, respectively. Higher magnification of the white box in (**A**), (**B**) and (**E**) are shown in (**B**), (**C**) and (**F**). Scale bar: 1000 μm in (**A**) and (**H**), 200 μm in (**B**), (**E**) and (**G**), 50 μm in (**C**) and (**F**). Negative control, mice with no *iCre* gene, is demonstrated in (**F**) and (**G**). (**D**) The percentage of tdTomato-positive neurons in NeuN-positive neurons was calculated at CA1, CA3 and DG, respectively (n = 6). The bars on each column represent the standard error of the mean. We assessed statistical differences using one-way ANOVA followed by Dunnett’s post hoc test. CA3 vs. CA1, *p* = 0.0375, CA3 vs. DG, *p* = 0.0078; *F*_(2,15)_ = 6.249, *p* = 0.0106.

In our previous study, western blot analysis detected endogenous BEGAIN in the olfactory bulb, midbrain, hippocampus, and cerebrum. Extremely high expression of BEGAIN was detected in the hippocampus^6^. BEGAIN-iCre::Ai9 reporter mice showed the distribution of BEGAIN-positive neurons in the forebrain including the hippocampus (Fig. 2A,B). The tdTomato was detected in NeuN-positive soma in the CA1 (16.00 ± 4.33) and DG (19.49 ± 6.15), but not in the CA3 (0.94 ± 0.24, Fig. 2B–D). The ratio in CA1 (*p* = 0.0375) and DG (*p* = 0.0078) is higher than that in DG (*F*_(2,15)_ = 6.249, *p* = 0.0106). In the spinal cord, tdTomato was highly expressed in the superficial laminae in the spinal dorsal horn (Fig. 2E,F), consistent with our previous immunohistochemical findings^6^. The BEGAIN-expressing neurons were identified in laminae II–III (Fig. 2F). The specificity of BEGAIN signals was verified by negative labeling in the Ai9 reporter mouse line (Fig. 2G,H).

### Detection of endogenous BEGAIN in the hippocampus

**Figure 3:**
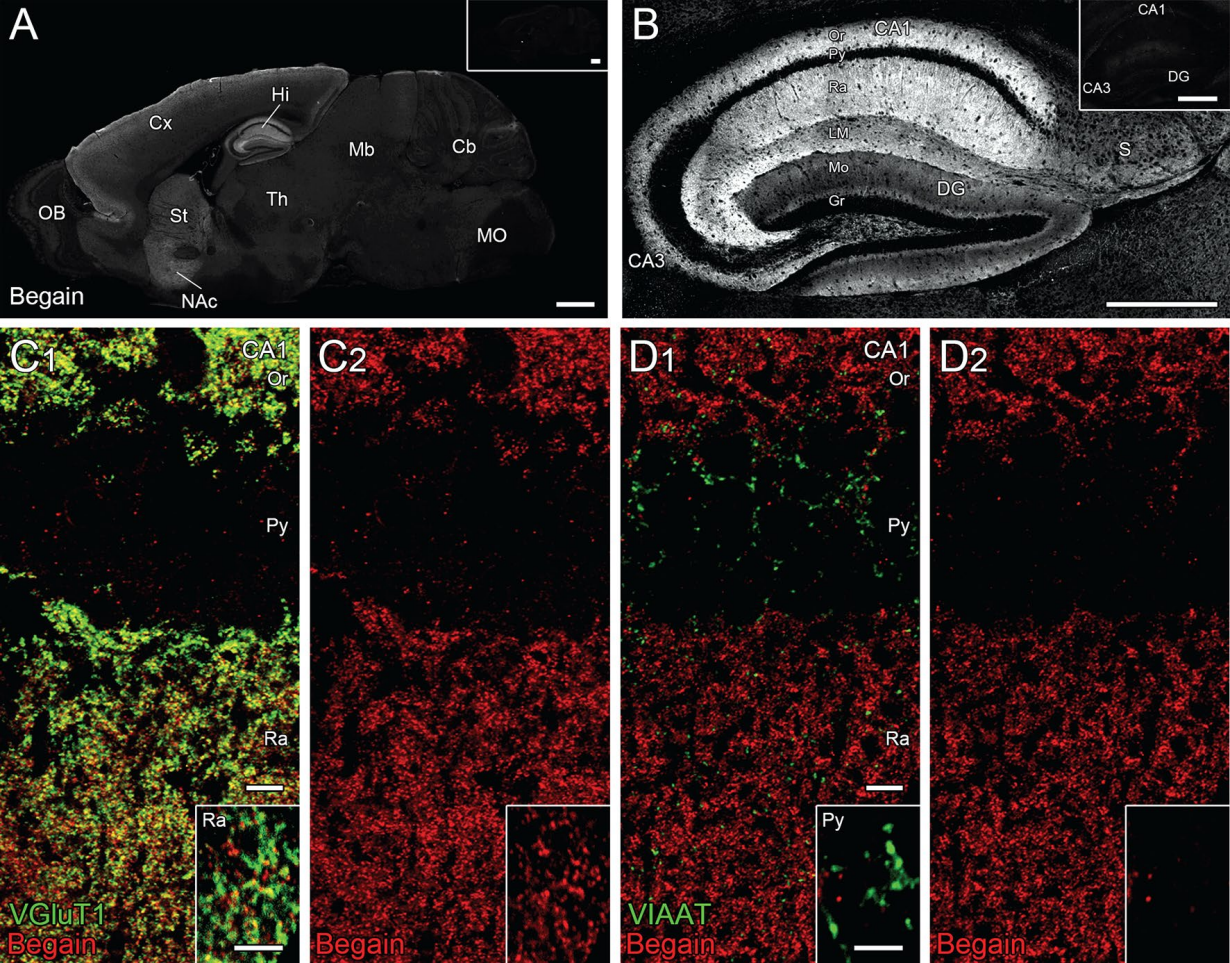
Expression of BEGAIN in the hippocampus. (**A**,**B**) Immunohistochemistry of brain-enriched guanylate kinase-associated protein (BEGAIN) with the anti-BEGAIN antibody. The absence of detection of BEGAIN in the BEGAIN-knockout mice is shown in the insets in A and B. Scale bar: 1000 μm in (**A**) and 500 μm in (**B**). (**C**,**D**) Double immunostaining with anti-BEGAIN and -VGluT1 or -VIAAT antibodies. A higher magnification of each is shown in the inset. Scale bar: 10 μm in (**C**) and (**D**) and 5 μm in the insets in (**C**) and (**D**).

Immunohistochemical analyses were performed to determine the distribution of BEGAIN in the brain. In our preliminary experiments, BEGAIN was not detected in the brain sections after of 4% paraformaldehyde (PFA) perfusion with and without antigen retrieval. Therefore, we used the fresh-frozen sections and performed antigen retrieval treatment, which resulted in immunohistochemical detection of endogenous BEGAIN widely in the brain. Relatively strong expression was detected in the cerebral cortex, striatum nucleus accumbens, and hippocampus (Fig. 3A), consistent with the distribution of the tdTomato fluorescence in BEGAIN-iCre::Ai9 mice (Fig. 2A). Different labeling patterns were also noted in some regions. In the hippocampus, immunohistochemical signals were high in the dendritic layers of the CA1-CA3 regions (Fig. 3B), while tdTomato fluorescence signals were intense for granule cells in the dentate gyrus and mossy fibers running in the CA3 region (Fig. 2B). Moreover, immunohistochemical signals were considerably low in the olfactory bulb, where intense signals for the tdTomato fluorescence were found in BEGAIN-iCre::Ai9 mice. The specificity of the immunohistochemical signals was verified by the lack of labeling in the BEGAIN-KO mice (insets in Fig. 3A,B).

### Synaptic localization of BEGAIN protein in the hippocampus

**Figure 4:**
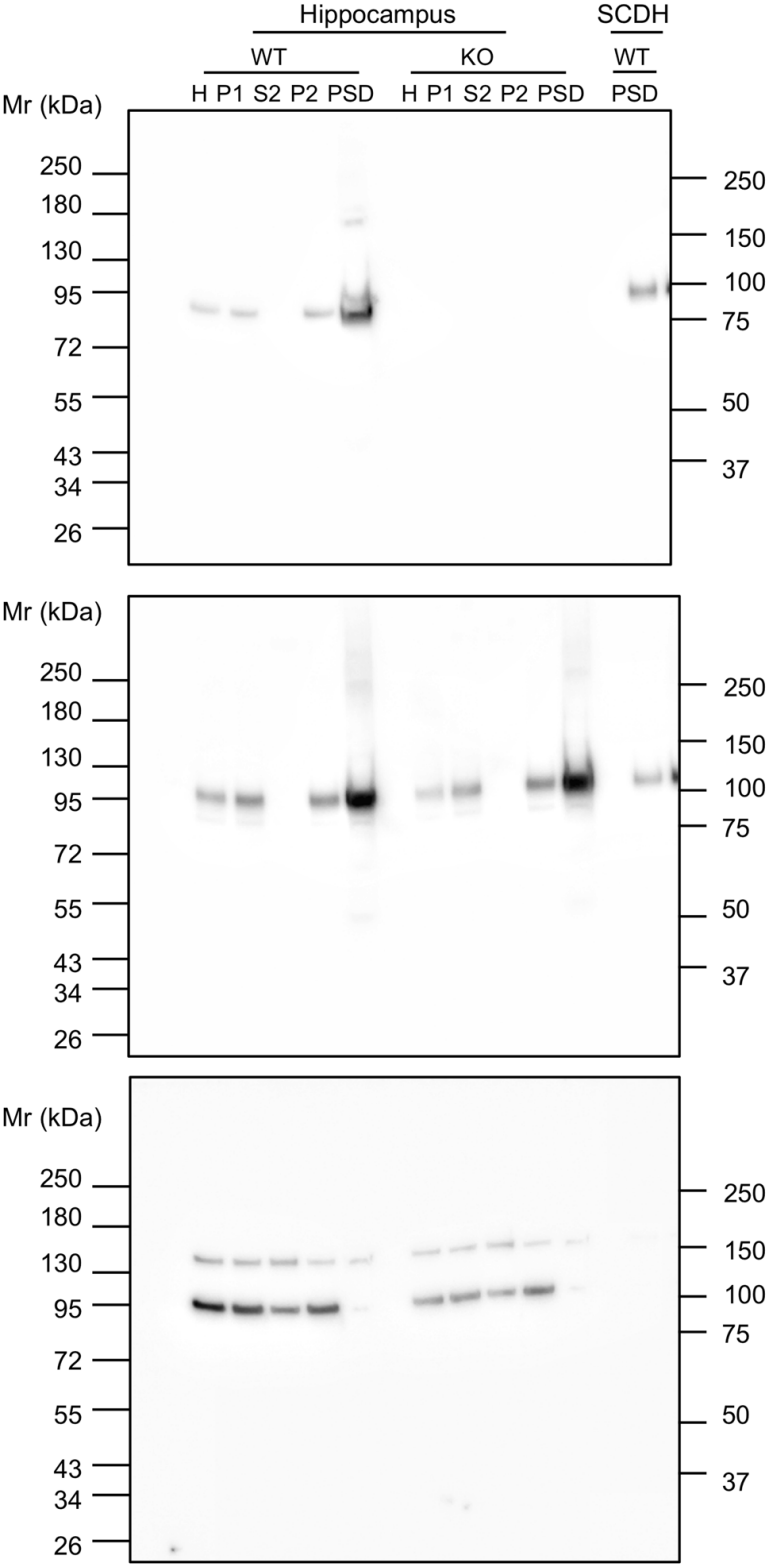
Distribution of BEGAIN in the PSD fraction. Western blot analysis of the subcellular fractions of mouse hippocampus and spinal dorsal horn (SCDH) from wild-type (WT) and brain-enriched guanylate kinase-associated protein (BEGAIN)-knockout (KO) mice. Total proteins (15 μg) reacted with the anti-BEGAIN (top), -PSD-95 (middle), and -vinculin (bottom) antibodies. *PSD* postsynaptic density.

**Figure 5:**
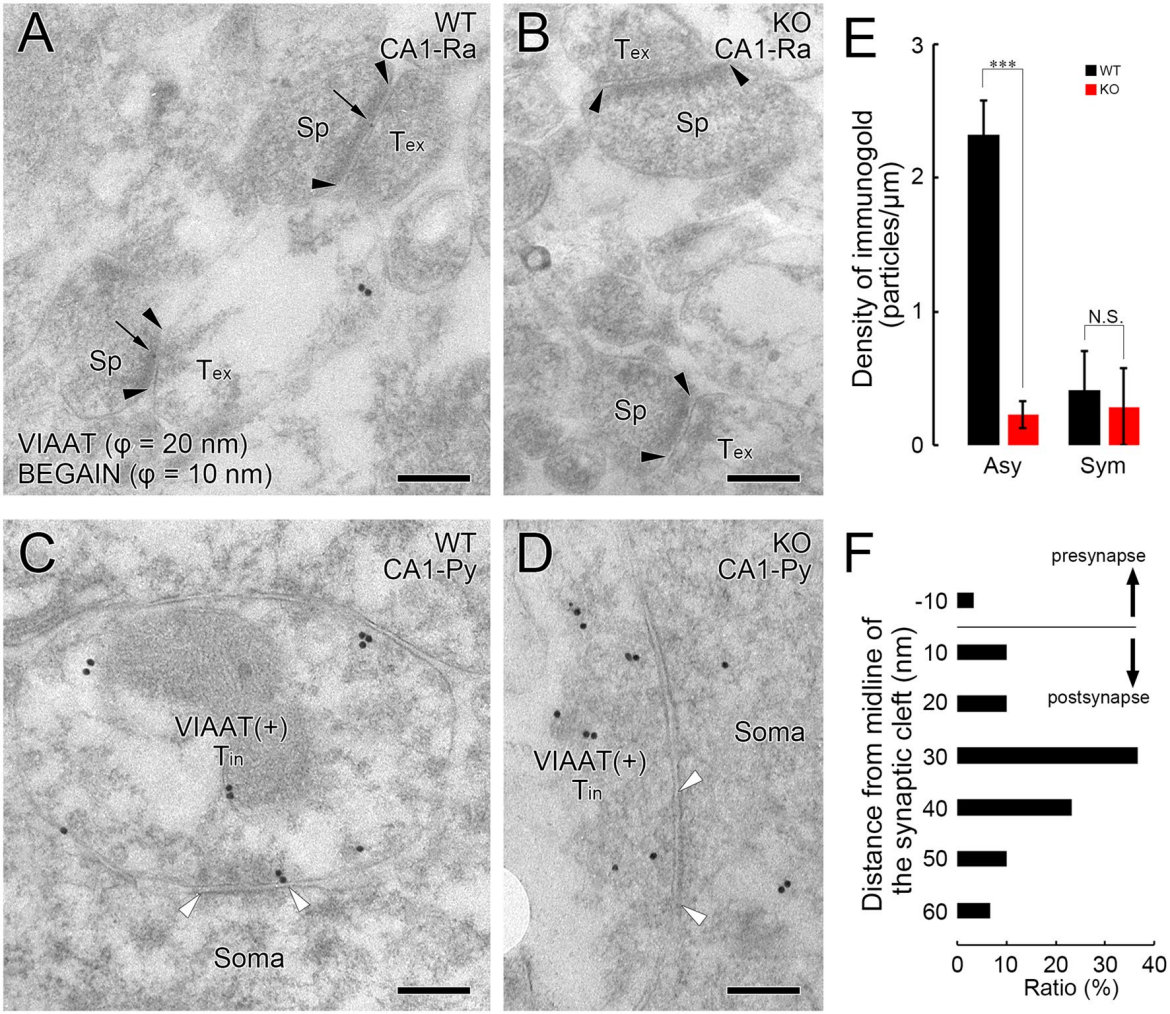
Synaptic localization of BEGAIN in the hippocampus. (**A**–**D**) Postembedding immunogold electron microscopy for brain-enriched guanylate kinase-associated protein (BEGAIN) (φ = 10 nm) and VIAAT (φ = 20 nm) in the stratum radiatum (**A**,**B**) and pyramidal cell layer (**C**,**D**) of wild-type (**A**,**C**) and BEGAIN knockout (**B**,**D**) mice. The edge of the postsynaptic specialization at asymmetrical (**A**,**B**) and symmetrical (**C**,**D**) synapses is indicated by a pair of black or white arrowheads, respectively. Scale bar: 200 nm. *Sp* spine, *Tex* excitatory presynaptic terminal, *Tin* inhibitory presynaptic terminal. (**E**) Compared with the background level, as determined using BEGAIN-KO mice (red columns), the density of immunogold labeling for BEGAIN in wild-type mice (black columns) is significantly higher at asymmetrical synapses (*p* < 0.001) but not at symmetrical synapses (*p* > 0.05). The analyzed synapses for BEGAIN are 130 or 111 at asymmetrical synapses and 25 or 20 at symmetrical synapses from wild-type (n = 2) and KO mice (n = 2), respectively. The bars on each column represent the standard error of the mean. ***p < 0.001; *N.S.* not significant; Mann–Whitney *U*-test. (**F**) Histograms showing the vertical distribution of BEGAIN at asymmetrical synapses (30 synapses from 2 mice). The distance was measured from the midline of the synaptic cleft to the center of an immunogold particle, in which minus and plus values represent nm on the presynaptic or postsynaptic side, respectively.

To determine whether BEGAIN was localized at excitatory, inhibitory, or both synapses in the dendritic area of CA, we performed double immunohistochemical analyses in the CA1 area with antibodies against VGluT1 and VIAAT, markers for the terminals of excitatory and inhibitory nerve endings, respectively (Fig. 3C,D). BEGAIN was well apposed to VGluT1-positive terminals (Fig. 3C), whereas BEGAIN signals were not associated with VIAAT-positive terminals (Fig. 3D). These results suggest that BEGAIN is selectively localized at excitatory synapses in the CA1. Western blot analysis with subcellular hippocampal fractions further confirmed that BEGAIN was highly concentrated in the PSD fraction, which was similar to PSD-95 as demonstrated by western blotting (Fig. 4). The PSD proteins are concentrated in the PSD to form a complex with receptors and signal transduction molecules and to orchestrate synapse transmission^1,3^. Thus, post-embedding immunoelectron microscopy for BEGAIN and VIAAT was performed to clarify the synaptic localization of BEGAIN in the hippocampus. Immunogold particles for BEGAIN were often detected along the PSD at VIAAT-negative asymmetrical synapses in the wild-type, but not in the BEGAIN-KO mice (Fig. 5A,B,E). On the other hand, no significant labeling was not detected along the post-synapse at VIAAT-labeled symmetrical synapses of both wild-type and BEGAIN-KO mice (Fig. 5C–E). Most immunogold particles for BEGAIN were distributed in the post-synaptic side from the midline of the synaptic cleft, peaking at 20–30 nm bin (Fig. 5F).

### Involvement of BEGAIN in learning and memory

**Figure 6:**
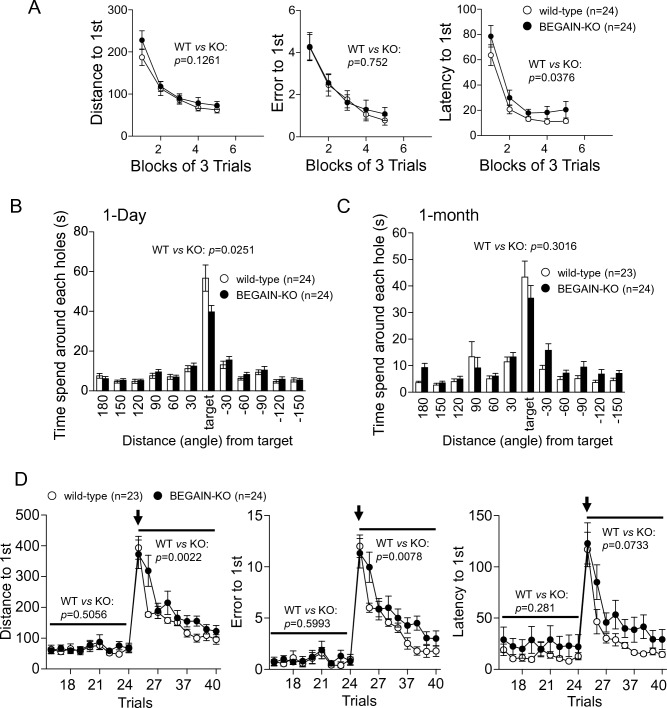
Impairment of spatial memory retention and reversal learning in the BEGAIN-KO mice in the Barns maze test. (**A**) Distance (left), error (middle) and latency (right) in the 1st trial (escape box). Data are presented as means of three trials. (**B**,**C**) Time spent around each hole in the probe tests conducted on day 1 (**B**) and at 1 month (**C**) after the last training trial. (**D**) Distance (left), error (middle) and latency (right) in the 1st trial after changing the escape box to the opposite as in reversal learning. The escape box was moved to the opposite location from the 25th trial (arrow). All values are presented as mean ± standard error of the mean. Data were analyzed using two-way repeated-measures analysis of variance (ANOVA). *p*-values indicate the genotype effect in two-way repeated measures ANOVA (**A**,**D**) and *t*-test with Bonferroni correction (target hole in **B**,**C**). *BEGAIN* brain-enriched guanylate kinase-associated protein, *KO* knockout.

To further investigate the contribution of BEGAIN to learning and memory, we conducted the Barnes circular maze test along with the contextual and cued fear conditioning test. In the learning process of the Barnes maze test, BEGAIN-KO mice showed slightly longer latency to 1st (escape box) than wild-type mice (genotype effect, *F*_(1,46)_ = 4.586, *p* = 0.0376; block of 3 trials effect, *p* < 0.0001; interaction genotype × block of 3 trials, *p* = 0.8717, Fig. 6A, right), but there were no significant differences in terms of the distance (genotype effect, *F*_(1,46)_ = 2.427, *p* = 0.1261; block of 3 trials effect, *p* < 0.0001; interaction genotype × block of 3 trials, *p* = 0.6391, Fig. 6A, left) and the error in the 1st trial (genotype effect, *F*_(1,46)_ = 0.101, *p* = 0.7520; block of 3 trials effect, *p* < 0.0001; interaction genotype × block of 3 trials, *p* = 0.9859, Fig. 6A, center). The probe test performed 24 h after the last training and show that both genotypes of mice learned the location of the target hole after these trials (One-way ANOVA; wild-type; *F*_(23,11)_ = 39.413, *p* < 0.0001, BEGAIN-KO; *F*_(23,11)_ = 43.128, *p* < 0.0001, Two-way repeated measures ANOVA; genotype effect, *F*_(1,46)_ = 1.023, *p* = 0.3170; hole effect, *p* < 0.0001; interaction genotype × holes, *p* < 0.0001, Fig. 6B). On the other hand, the time spent around the target hole by the BEGAIN-KO mice was significantly shorter than that by the wild-type mice (genotype effect, *t*_(46)_ = 2.315, *p* = 0.0251, Fig. 6B). The probe test also performed 1 month after the last training and showed that mice of both genotypes retained memory of the target hole location (One-way ANOVA; wild-type; *F*_(22,11)_ = 18.332, *p* < 0.0001, BEGAIN-KO; *F*_(23,11)_ = 13.392, *p* < 0.0001, Two-way repeated measures ANOVA; genotype effect, *F*_(1,45)_ = 3.613, *p* = 0.0637; hole effect, *p* < 0.0001; interaction genotype × holes, *p* = 0.1792, Fig. 6C). The BEGAIN-KO mice spent slightly less time around the target hole than the wild-type mice, but this was not significant at 1 month after the last training session (genotype effect, *t*_(45)_ = 1.045, *p* = 0.3016, Fig. 6C). In addition, Reversal learning was also established in both genotypes of mice, but BEGAIN-KO mice showed an increase in distance (genotype effect, *F*_(1,43)_ = 10.579, *p* = 0.0022; trial effect, *p* < 0.0001; interaction genotype × trials, *p* = 0.0937, Fig. 6D, left), latency (genotype effect, *F*_(1,43)_ = 3.370, *p* = 0.0733; trial effect, *p* < 0.0001; interaction genotype × trials, *p* = 0.6170, Fig. 6D, right) and error in the 1st trial (genotype effect, *F*_(1,43)_ = 7.798, trial effect, *p* < 0.0001; interaction genotype × trials, *p* = 0.1707, *p* = 0.0078, Fig. 6D, center) after the change to the opposite side of the escape box. These results indicate that BEGAIN is implicated in the spatial reference memory and reversal learning.

**Figure 7:**
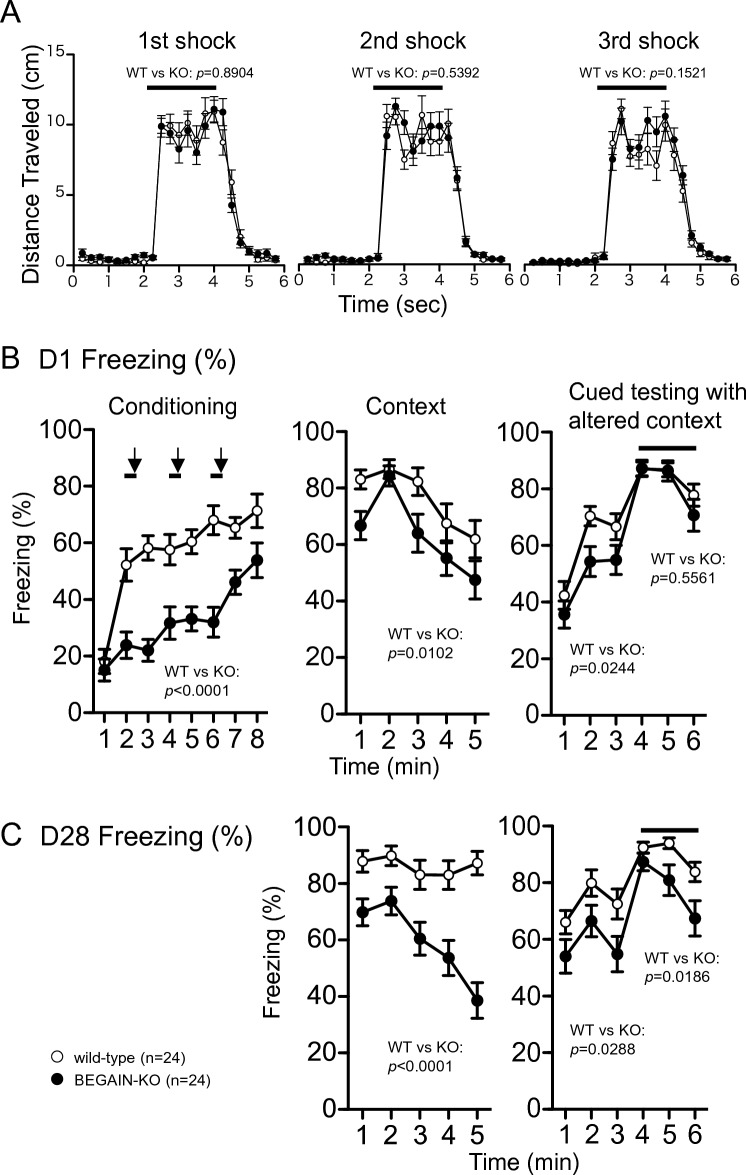
Impairment of retention of contextual memory in the BEGAIN-KO mice in the contextual cued fear conditioning test. (**A**) Total distance travelled (cm) in 6 s with electrical stimulation (2 s, black bar) for conditioning. The statistical analysis was carried out in the six seconds indicated. (**B**) Freezing responses by electrical shock (arrow) with cue tone (black bar) on the conditioning day (left; conditioning). Freezing responses in the contextual test on day 1 after conditioning (middle; context). Freezing responses in the cue test on day 1 after conditioning (right). (**C**) Freezing responses in the contextual test on day 28 after conditioning (left). Total distances in the cue test on day 28 after conditioning (right). Bold lines represent tone [right in (**B**) and right in (**C**)]. All values are presented as mean ± standard error of the mean. *p*-values indicate the genotype effect in two-way repeated-measures analysis of variance (ANOVA). Analyses were performed separately for pre-tone (1–3′) and cue-tone (4–6′) periods [right in (**B**) and right in (**C**)].

BEGAIN-KO mice showed a shorter freezing ratio than the wild-type mice during the conditioning period in the contextual and cued fear conditioning test. This difference was more pronounced up to the third electric shock, after which BEGAIN-KO mice also showed an increase in freezing ratio (genotype effect, *F*_(1,44)_ = 42.721, *p* < 0.0001; time effect, *p* < 0.0001; interaction genotype × time, *p* = 0.0036, Fig. 7B, left). However, the distance traveled by the BEGAIN-KO mice following the electric shock was not significantly different from that by the wild-type mice (genotype effect of the first shock, *F*_(1,44)_ = 0.019, *p* = 0.8904; time effect, *p* < 0.0001; interaction genotype × time, *p* = 0.7124; second shock, *F*_(1,44)_ = 0.383, *p* = 0.5392; time effect, *p* < 0.0001; interaction genotype × time, *p* = 0. 2914; third shock, *F*_(1,44)_ = 2.124, *p* = 0.1521; time effect, *p* < 0.0001; interaction genotype × time, *p* = 0.5871, Fig. 7A). This was in agreement with the finding of our previous study, which showed that BEGAIN-KO did not alter the sensitivity to nociceptive stimuli under normal conditions^6^. After conditioning, the low freezing ratio was also demonstrated in the contexed testing at 1 day (genotype effect, *F*_(1,44)_ = 7.209, *p* = 0.0102; time effect, *p* < 0.0001; interaction genotype × time, *p* = 0. 5253, Fig. 7B, middle) and 28 days after conditioning (genotype effect, *F*_(1,44)_ = 27.504, *p* < 0.0001; time effect, *p* < 0.0001; interaction genotype × time, *p* = 0.0003, Fig. 7C, left), and this phenotype was more pronounced after 28 days. These results suggest that BEGAIN is associated with contextual fear conditioning that associates chambers with electric shocks and their retention. In the cued testing with altered context, BEGAIN-KO mice demonstrated a lower freezing ratio before cue tone at 1 day (genotype effect for 1–3 min, *F*_(1,44)_ = 5.432, *p* = 0.0244; time effect, *p* < 0.0001; interaction genotype × time, *p* = 0.4909; Fig. 7B, right) and 28 days after conditioning (genotype effect for 1–3 min, *F*_(1,44)_ = 5.112, *p* = 0.0288; time effect, *p* = 0.0011; interaction genotype × time, *p* = 0.7088). Moreover, significant differences in freezing were detected with cue tone at 28 days (genotype effect for 4–6 min, *F*_(1,44)_ = 5.971, *p* = 0.0186; time effect, *p* < 0.0001; interaction genotype × time, *p* = 0.1083, Fig. 7C, right), but not at 1 day after conditioning (genotype effect for 4–6 min, *F*_(1,44)_ = 0.352, *p* = 0.5561; time effect, *p* < 0.0001; interaction genotype × time, *p* = 0.3377, Fig. 7B, right). These results suggest that BEGAIN is not involved in cued fear conditioning that associates sounds (cued tones) with electric shocks, but it is associated with the retention of cued fear memory.

## Discussion

We demonstrated that BEGAIN is widely distributed in the brain and highly expressed in the hippocampus. In addition, it was found that BEGAIN was distributed in the dendritic area, such as orient, radial and molecular layers in CA (Fig. 3A,B), with some co-localizing with NeuN (Sup. 1). Moreover, BEGAIN was observed in the molecular layer of DG; however, the level of expression was lower than that in the CA (Fig. 3B). On the other hand, the signal of tdTomato in the granule cell layer, mossy fibers and bright layer was stronger than that in the orient, radial and molecular layers (Fig. 2B,E). This may be due to the fact that transport and degradation of BEGAIN and tdTomato are regulated by different mechanisms. Furthermore, *BEGAIN* messenger (m)-RNA was also detected in all the neurons in the brain and spinal cord in our experiments (data not shown) and in the Allen brain atlas (https://mouse.brain-map.org/gene/show/119936) by in situ hybridization. The tdTomato in BEGAIN-iCre::Ai9 mice was specifically recognized in the superficial layer of the spinal cord, but not in the ventral horn (Fig. 2C,D), which was in accordance with our previous observation by immunostaining with the anti-BEGAIN antibody^6^. These results infer that *BEGAIN* mRNA expression in the CNS is ubiquitous, but protein expression is limited. This indicates that translation, degradation, and localization of BEGAIN protein in the CNS may be regulated in a region-dependent manner. Further elucidation of these regulations may help to understand the tissue- and site-specific functions of BEGAIN.

Several receptors, scaffold proteins, and other signal transduction molecules concentrate and form the protein complex in the PSD, such as the NMDAR complex^21,22^. The architecture of the PSD has been reported to be dynamic in response to synaptic activities such as learning and memory^5,23^. PSD-95 is an abundant protein in the PSD, which binds directly to NMDARs via its PDZ domain^2^. PSD-95 also forms multimeric complexes by itself and binds directly to other PSD proteins, thus contributing to the formation of giant protein complexes in the PSD^2,11^. Since BEGAIN forms a homodimer in the N-terminal region and interacts with the GK domain of PSD-95 in the C-terminal region^7,24^, BEGAIN is also expected to act as a molecule contributing to complex formation in the PSD. In addition, we demonstrated that BEGAIN is localized at the asymmetrical synapses in the hippocampus. More than 60% of immunogold particles for BEGAIN were located within 30–40 nm from the post-synaptic membrane (Fig. 5). GluN2 and PSD-95 are located 10–12 nm from the postsynaptic membrane, and disk large-associated protein 1 (DLGAP1/GKAP/SAPAP1), which binds to the GK domain of PSD-95 like BEGAIN, is located approximately 25–27 nm from the postsynaptic membrane^25^. Thus, our results indicate that BEGAIN is a component of the PSD protein complex. Deletion of PSD-95 leads to a reduction in the number of synapses containing glutamate receptors and defects in NMDAR transmission^26^. Similarly, removing GKAP also leads to the loss of AMPAR-containing synapses and weakened synaptic transmission^27,28^. In addition, BEGAIN-KO mice exhibited a relatively longer delay of EPSCs between times to peak for the NMDA receptor in the spinal dorsal horn after nerve injury, which may have caused a reduction in the number of activated NMDAR during glutamatergic synaptic events in neuropathic pain^6^. Therefore, these previous reports together with our results suggest that BEGAIN modulates the number of synapses via NMDARs activity. In contrast, the GluN2 subunits of the hippocampal cPSD fraction of untreated (naive) BEGAIN-KO mice was slightly but not significantly reduced in comparison with that of wild-type mice (Sup. 2). This may have been because the effect of BEGAIN on NMDAR localization is partially dependent on synaptic plasticity during learning and memory. In the present study, we demonstrated the role of BEGAIN in learning and memory by behavioral analyses using BEGAIN-KO mice. In future electrophysiological analyses of both early and late phases of LTP and LTD using BEGAIN-KO mice, we will clarify the direct relationship of BEGAIN with protein distribution, synaptic plasticity and memory formation.

In this study, we clarified that BEGAIN was highly concentrated in the hippocampus and specifically localized at the asymmetrical synapses in the CA1 (Figs. 4 and 5), suggesting that BEGAIN may be involved in hippocampal function. We also showed that learning and memory were impaired by BEGAIN deficiency (Figs. 6 and 7). The memory retention for reference memory at 1 day after training and the fear memory (contextual fear conditioning and cued fear conditioning) at 28 days after conditioning was impaired in the BEGAIN-KO mice in comparison with the wild-type mice (Figs. 6 and 7). Retention of reference working memory was still reduced in BEGAIN-KO mice after 1 month of training, but the significant difference between wild-type and BEGAIN-KO mice observed at 1 day was not observed after 1 month (Fig. 6C). On the basis of the results of these behavioral analyses, it can be inferred that the effects of BEGAIN on long-term memory may be greater in fear memory than in spatial reference memory long-term. On the other hand, the highest expression of BEGAIN was confirmed in the hippocampus (Figs. 2 and 3). The hippocampus has been reported to be important not only for both reference and fear memories^29^. In addition, extensive expression of BEGAIN, including in the amygdala, was also demonstrated (Fig. 1). Further analysis of the diverse behaviors and region-specific BEGAIN-interacting molecules is needed to understand the higher brain functions of BEGAIN. These analyses may also elucidate why the effect of reference memory was modest in BEGAIN-KO mice (Fig. 6C) and why there was less freezing during initial conditioning (Fig. 7B).

The molecular mechanisms of chronic pain and memory share similarities, and many molecules are known to be involved in the plasticity of both, including PSD proteins such as glutamate receptors, kinases, and scaffold proteins, whose activities and interactions via post-translational modifications have been reported^14,15,17,18,30–32^. NMDARs are common important molecules for synaptic plasticity in the spinal cord and hippocampus. Mechanical allodynia, a symptom of neuropathic pain, was suppressed by the NMDAR antagonist APV and the GluN2B-specific antagonist Ro25-6981^6,33,34^. In addition, chronic intraventricular infusion of APV impairs spatial learning and LTP in pyramidal neurons^31^. Furthermore, fear-related memory retention was impaired in the GluN2B Y1472F-KI mice^14^, and mechanical allodynia was also attenuated in the KI mice^6^. In the present study, we demonstrated the impairment of learning and memory (Figs. 6 and 7) and attenuation of mechanical allodynia in the BEGAIN-KO mice^6^. The findings of the abovementioned studies along with our results suggest that BEGAIN, like GluN2B-NMDAR, is involved in synaptic plasticity for both learning and memory and neuropathic pain. Additionally, conditional knockout of GluN1 in CA1 and DG has been reported to impair reversal learning, indicating that NMDAR activity is involved in reversal learning^35,36^. BEGAIN localizes to the PSD at glutamatergic synapses via direct binding to PSD-95 (Fig. 5)^7,25^. Furthermore, inhibition of NMDAR reportedly suppresses BEGAIN expression in PSDs in the spinal dorsal horn and at the plasma membrane in the hippocampal neurons^6,24^, suggesting that BEGAIN may accumulate in PSDs during the activation of NMDAR. Moreover, BEGAIN-KO mice also showed impairment of reversal learning (Fig. 6D). Considering these reports and our findings, it can be inferred that BEGAIN may interact with PSD complexes in the hippocampus and contribute to reversal learning via NMDAR activity. Thus, we conclude that BEGAIN is associated with physiological and pathological states, such as learning and memory and neuropathic pain.

## Materials and methods

### Animals

BEGAIN-KO mice were generated using a gene targeting technique as previously reported^6^. BEGAIN-Cre-3 × Venus mice were generated using the RENKA embryonic stem (ES) cell line derived from the C57BL/6N strain^37^. iCre and three tandem Venus genes (3 × Venus) were inserted into the second exon of the *Begain* gene (Ex2, Fig. 1A). Homologous recombination of the gene between ES cells was detected using Southern blot analysis. Ai9 mice (B6. Cg-Gt (ROSA) 26Sor tm9 (CAG-tdTomato) Hze; Jackson Labs, Stock #007909) were obtained from the Jackson Laboratory. All procedures described here were reviewed and approved by the Animal Care and Use Committee of Kansai Medical University and National Institute for Physiological Sciences in Japan, and experiments were performed under the institutional guiding principles for the care and use of laboratory animals. All methods were performed in accordance with the ARRIVE guidelines (https://arriveguidelines.org).

### Antibodies

Rabbit anti-BEGAIN against C-terminal 17 amino acid of mouse BEGAIN was used^6^. Commercially available antibodies against PSD-95 (Abcam, Upstate Biotechnology), vinculin (Abcam), VGluT1 (guinea pig, Nittobo Medical), VIAAT (guinea pig, Nittobo Medical), and NeuN (Millipore) were also used.

### Subcellular fractionation of the hippocampus

After anesthesia with isoflurane, 20–29 male wild-type (WT) and BEGAIN-KO mice (7–12 weeks old) were killed, and their hippocampi were collected (approximate wet weight 1.0 g). The hippocampus was homogenized in solution A [0.32-M sucrose, 1 mM NaHCO_3_, 1-mM MgCl_2_, 0.5-mM CaCl_2_, PhosSTOP (Roche), and protease inhibitor cocktail (Nacalai Tesque)] with a Potter–Elvehjem homogenizer. After centrifugation of the homogenate at 800×*g* for 10 min, the pellet (P1; nuclear) and supernatant (S1) were separated. The S1 fraction was centrifuged at 13,800×*g* for 20 min. The supernatant (S2) and precipitate (P2) were collected as the cytosolic and membrane fractions. The PSD fraction was prepared as described by Carlin et al. with slight modifications^22^. The P2 fraction was then suspended in solution B (0.32 M sucrose containing 1-mM NaHCO_3_) and applied onto a discontinuous sucrose gradients composed of 3.4 ml of 1.2-M, 3.4 ml of 1.0-M, and 3 ml of 0.85-M sucrose in 1-mM NaHCO_3_ in tubes. The tube was centrifuged at 82,500×*g* for 120 min. The interface between 1.0- and 1.2-M sucrose was collected and dissolved for 15 min with buffer C consisting of 0.5% Triton X-100 and 6-mM Tris–HCl (pH 8.0). For collection of the insoluble fraction, the interface fraction was centrifuged at 32,800×*g* for 30 min. The insoluble fraction was dissolved in 7-M urea, 2-M thiourea, 4% CHAPS, and 2% SDS and used as the PSD fraction.

### Western blot analysis

All subcellular fractions from the hippocampus were subjected to a single gel for SDS-PAGE (4–12% acrylamide), and the separated proteins were transferred to a polyvinylidene fluoride membrane. After blocking for 1 h at room temperature (25 °C) with 3% skim milk in TBS-T buffer consisting of 0.1% Triton X-100, 150-mM NaCl and 10-mM Tris–HCl (pH 7.5), the membrane was incubated at 4 °C overnight with anti-BEGAIN (1 μg/ml), anti-PSD-95 (1:1000) and anti-vinculin (1:10,000) antibodies. The membrane was washed with the TBS-T buffer and incubated for 1 h with horseradish peroxidase-conjugated goat anti-rabbit IgG (1:20,000; Invitrogene) or goat anti-mouse IgG (1:20,000; GE Healthcare). It was then washed four times with TBS-T buffer. Immunoreactivity was detected by using an enhanced chemiluminescence detection kit (Chemi-Lumi One Super; Nacalai Tesque). Detection of several proteins, such as BEGAIN, PSD-95 and vinculin, in a single gel was performed sequentially. That is, the preceding antibody was stripped from the polyvinylidene fluoride membrane, which was then re-probed with other primary antibodies for the detection of the next protein.

### Histochemistry

Animals were anesthetized by an intraperitoneal administration of sodium pentobarbital (50 mg/kg) and perfused with 4% paraformaldehyde (PFA) in 0.12-M sodium phosphate (PB, pH 7.4) for immunofluorescence for NeuN and tdTomato and 1% PFA in PB for post-embedding immunogold electron microscopy. After dissection, brain and spinal cord tissues were postfixed for 4 h in the same fixative at 4 °C and cryoprotected overnight in 30% (w/v) sucrose in PB. Sagittal and coronal sections of the brain and transverse sections of the spinal cord were cut on a cryostat or microtome (14 or 40 μm thick) and processed for immunohistochemistry with the anti-NeuN antibody (1:500). For the detection of endogenous BEGAIN in the mouse brain, fresh-frozen sections were cut on a cryostat (20 μm). The sections were stained with anti-BEGAIN, anti-VGluT1 and anti-VIAAT antibodies (1 μg/ml each) as primary antibodies after fixation with 4% PFA in PB for 15 min and antigen retrieval at 95 °C for 30 min in Immnosaver solution (Nisshin). Sections were then incubated with Alexa Fluor 488 goat anti-mouse IgG as secondary antibody (1:300, Invitrogen) or with a mixture of Alexa 488- and Cy3-labeled species-specific secondary antibodies (1:200 dilution; Invitrogen; Jackson ImmunoResearch) for 1 to 2 h at room temperature. Images for fresh-frozen sections were captured using a confocal laser scanning microscope equipped with 473 and 559 diode laser lines, and UPlanSApo (10 ×/0.40), UPlanSApo (20 ×/0.75) and PlanApoN (60 ×/1.4, oil immersion) objective lenses (FV1200; EVIDENT). Fluorescence images of tdTomato were captured using the Zyla4.2 sCMOS camera (Andor) mounted onto an EVIDENT IX81 (EVIDENT) and DragonFly500 spinning disk confocal imaging system (Oxford Instruments). For quantitative analysis, histochemical data were analyzed with Imaris software (Oxford Instruments); NeuN- and tdTomato-positive neurons were detected with the Spot tool, and regions of interest, CA1, 3, and DG, were created with the Surface tool. The percentages of co-localized neurons in CA1, CA3, and DG were analyzed by one-way ANOVA followed by Dunnett’s post hoc test.

### Electron immunohistochemistry

For post-embedding immunogold electron microscopy, microslicer sections (400 μm) were cryoprotected by immersion in 30% glycerol in PB for 30 min, and then frozen rapidly in liquid propane in an EM CPC unit (Leica Microsystems). Frozen sections were immersed in 0.5% uranyl acetate in methanol at − 90 °C in an AFS freeze-substitution unit (Leica Microsystems), infiltrated at − 45 °C with Lowicryl HM-20 resin (Electron Microscopy Sciences), which was then polymerized with ultraviolet light. Ultrathin sections were cut using an Ultracut ultramicrotome (Leica Microsystems) and mounted on nickel grids.

Ultrathin sections on nickel grids were etched with saturated sodium-ethanolate solution for 1–5 s and treated with successive solutions, as follows: sections were incubated in 50-mM glycine in an incubation solution [0.01% Triton X-100 in Tris-buffered saline, pH 7.4 (TTBS)] for 10 min. For double labeling against VIAAT and BEGAIN, sections were incubated in a blocking solution containing 2% normal goat serum, followed by the guinea pig anti-VIAAT antibody (20 μg/ml) diluted in 2% normal goat serum in TTBS overnight and then colloidal gold-conjugated (20 nm) anti-guinea pig IgG in the blocking solution for 2 h. After extensive washing in distilled water, sections were incubated in a blocking solution containing 2% normal guineapig serum (Nichirei) in TTBS for 10 min, rabbit anti-BEGAIN antibody diluted with 2% normal guinea pig serum in TTBS overnight, and colloidal gold-conjugated (10 nm) anti-rabbit IgG (1:100, British BioCell International) in a blocking solution for 2 h. After extensive washing in distilled water, sections were fixed with 2% OsO_4_ for 15 min, and stained with 5% uranyl acetate/40% ethanol for 90 s and Reynold’s lead citrate solution for 60 s. Photographs were taken from the molecular layer of the cerebellum with an H-7100 electron microscope (Hitachi).

For quantitative analysis, the density of post-synaptic membrane-associated immunogold particles, which were defined as those < 20 nm from the cell membrane, were counted on electron micrographs and analyzed using MetaMorph software (Molecular Devices). The vertical distribution at synapses was examined by sampling synaptic profiles whose presynaptic and post-synaptic membranes were cut perpendicularly to the plane of the synaptic cleft, and by measuring the distance from the midline of the synaptic cleft to the center of an immunogold particle.

### Behavioral studies

Behavioral tests were carried out with 24 wild-type and 24 BEGAIN-KO male mice, which were obtained by heterozygous intercrossing breeding. The mice used in the study were 4-to-5-week-old mice and were housed in groups of four (two pairs of wild-type and BEGAIN-KO mice) per cage in a room with a 12-h light/dark cycle and with access to food and water ad libitum. Behavioral testing was performed between 9:00 a.m. and 6:00 p.m. The raw data from the behavioral test and the information about each mouse are available on the public database “Mouse Phenotype Database” (http://www.mouse-phenotype.org/).

### Barnes circular maze test

The maze apparatus consisted of a white circular disk 1.0 m in diameter with 12 holes equally spaced around the perimeter, which was elevated 75 cm from the floor. A black escape box (17 × 13 × 7 cm) was located under one of the holes as the target (O’Hara & Co.). The maze was rotated daily, with the spatial location of the target unchanged with respect to the distal visual room cues, to prevent a bias based on olfactory or the proximal cues within the maze. Three trials per day were conducted every day until the total number of trials reached 15. After 1 day and 1 month, a probe trial without the escape box was conducted to confirm that this spatial task was acquired based on navigation by distal environment room cues. One mouse received a different exposure in the second probe test and was excluded from subsequent analyses for accuracy because of technical problems with the equipment.

### Contextual and cued fear conditioning test

Each mouse was placed in a transparent acrylic chamber (33 × 25 × 28 cm) with a stainless-steel grid floor (0.2-cm diameter, spaced 0.5 cm apart; O’Hara & Co.) and was allowed to explore freely for 2 min. Subsequently, a 55-dB white noise, which served as the conditioning stimulus (CS), was presented for 30 s. During the last 2 s of CS presentation, a mild foot shock (0.3 mA, 2 s), which served as the unconditioned stimulus (US), was presented. Two more CS-US pairings were presented with a 2-min inter-stimulus interval. Twenty-four hours after the conditioning, a context test was conducted in the same chamber. A cued test with altered context was then performed in a triangular chamber (33 × 29 × 32 cm) made of white opaque plastic, which was located in a different room. In each test, the freezing percentage and distance traveled were calculated automatically by using ImageFZ software (see section, “Image analysis and statistical analysis for behavioral tests”). Data are presented as mean ± SEM.

### Image analysis and statistical analysis for behavioral tests

The application software used for the behavioral studies was based on the public domain NIH Image program (developed at the United States National Institutes of Health and available at http://rsb.info.nih.gov/nih-image/) and ImageJ program (http://rsb.info.nih.gov/ij/), which were modified for each test (available through O’Hara & Co.). ImageFZ^38^ is freely available at the following URL: http://www.mouse-phenotype.org/software.html. Statistical analyses for behavioral studies were conducted with StatView (SAS Institute). Statistical tests used were two-way repeated measures ANOVA between genotypes for Figs. 6A–D and 7A–C, one-way ANOVA in each genotype for Fig. 6B,C, and *t*-test with Bonferroni correction as post hoc test between genotypes for Fig. 6A–C. *p* values less than 0.05 were considered statistically significant. Actual *F* and *p* values are given in the results, figures and supplementary table. Data are presented as mean ± standard error of the mean (SEM).

### Supplementary Information

Supplementary Information 1.

Supplementary Information 2.

Supplementary Information 3.

Supplementary Information 4.

Supplementary Table.

## Data Availability

The row data of the behavioral tests are accessible on the public database “Mouse Phenotype Database” (http://www.mouse-phenotype.org/).
