# Supplementary figures and images for: Brain-enriched guanylate kinase-associated protein, a component of the post-synaptic density protein complexes, contributes to learning and memory
Source: Sci Rep. 2023 Dec 12;13:22027. doi: 10.1038/s41598-023-49537-9 (PMC10716515; doi:10.1038/s41598-023-49537-9)

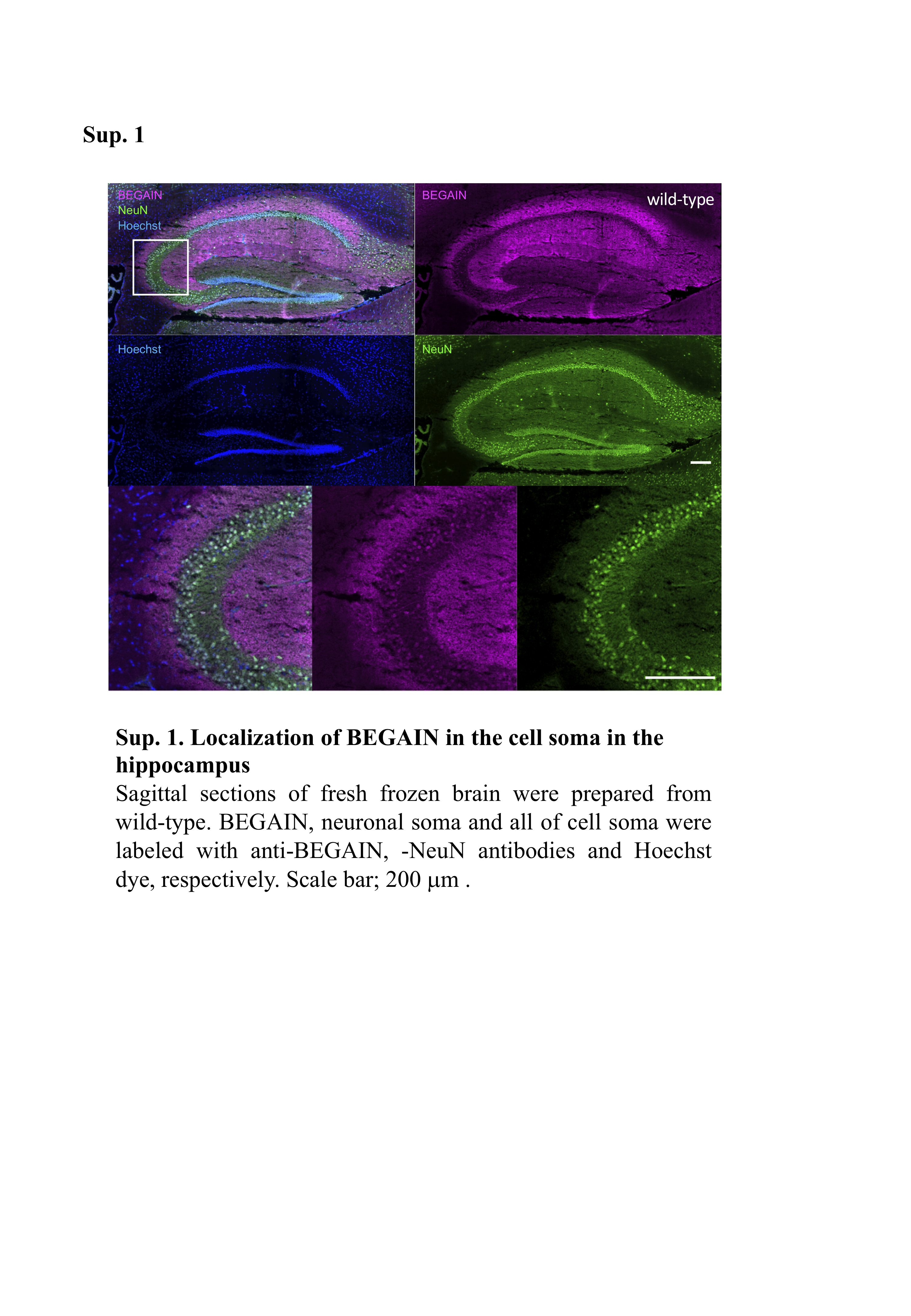

Supplement: Supplementary file 1 — Supplementary Information 1. [file 41598_2023_49537_MOESM1_ESM.tiff]

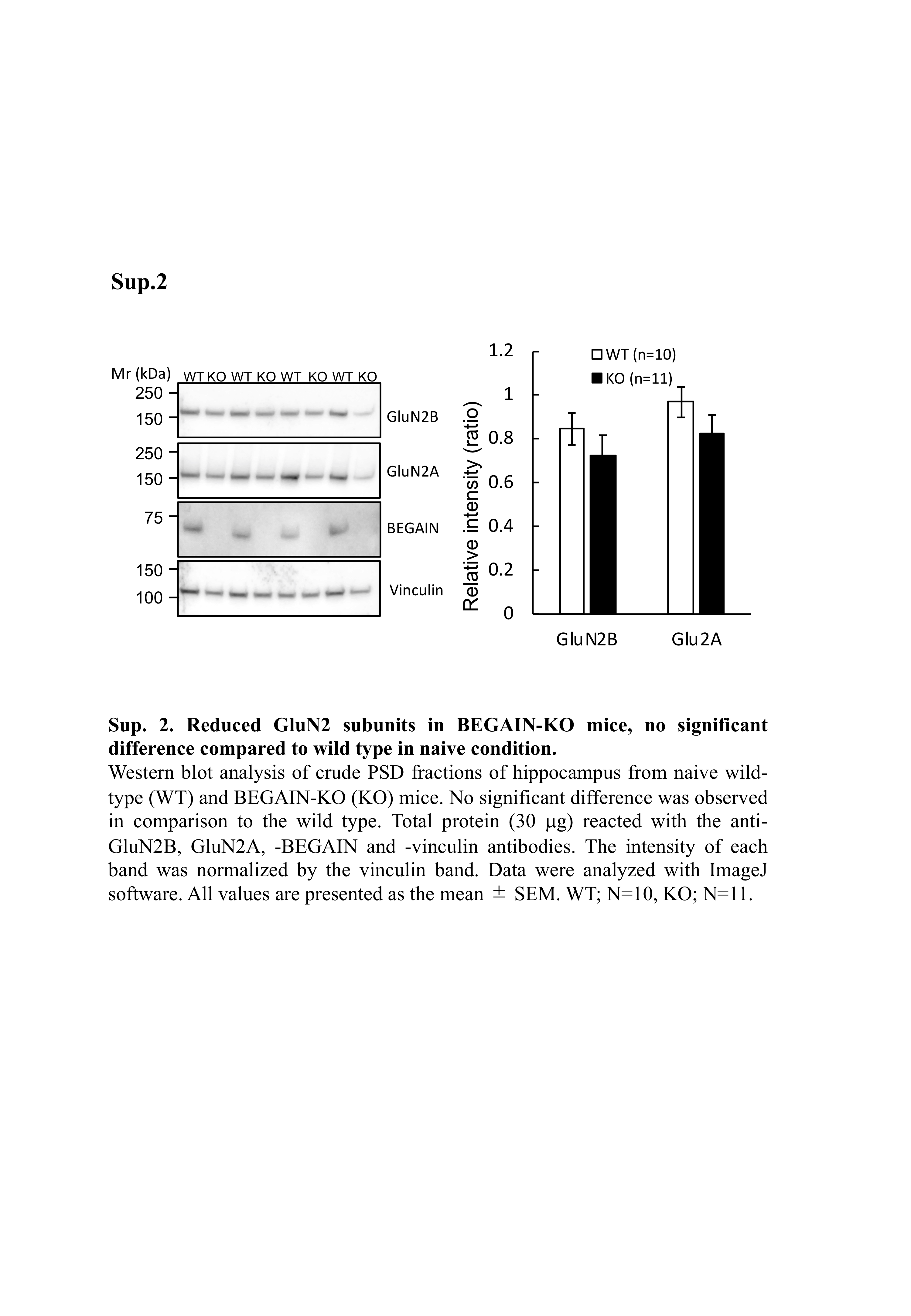

Supplement: Supplementary file 2 — Supplementary Information 2. [file 41598_2023_49537_MOESM2_ESM.tiff]

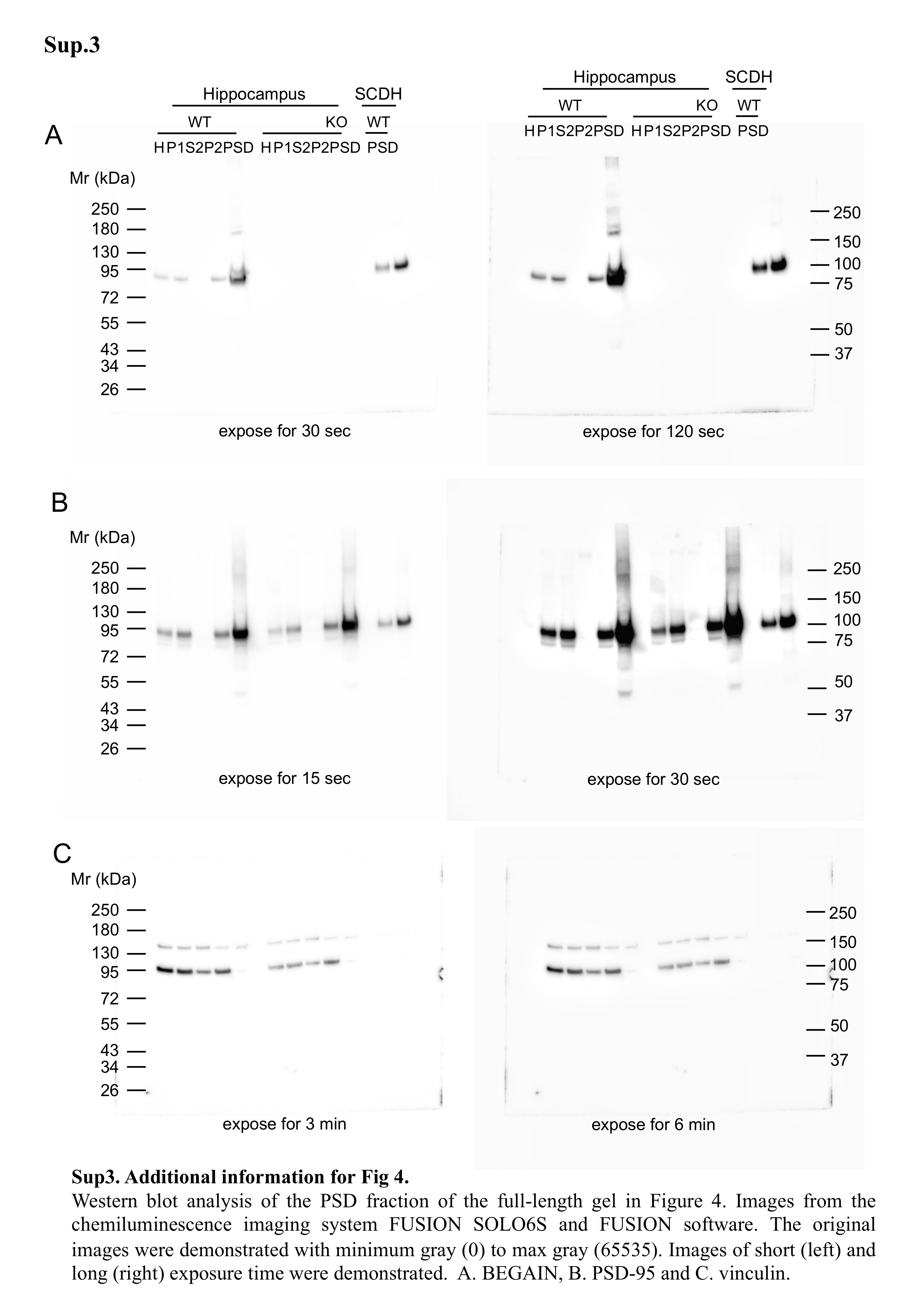

Supplement: Supplementary file 3 — Supplementary Information 3. [file 41598_2023_49537_MOESM3_ESM.tiff]

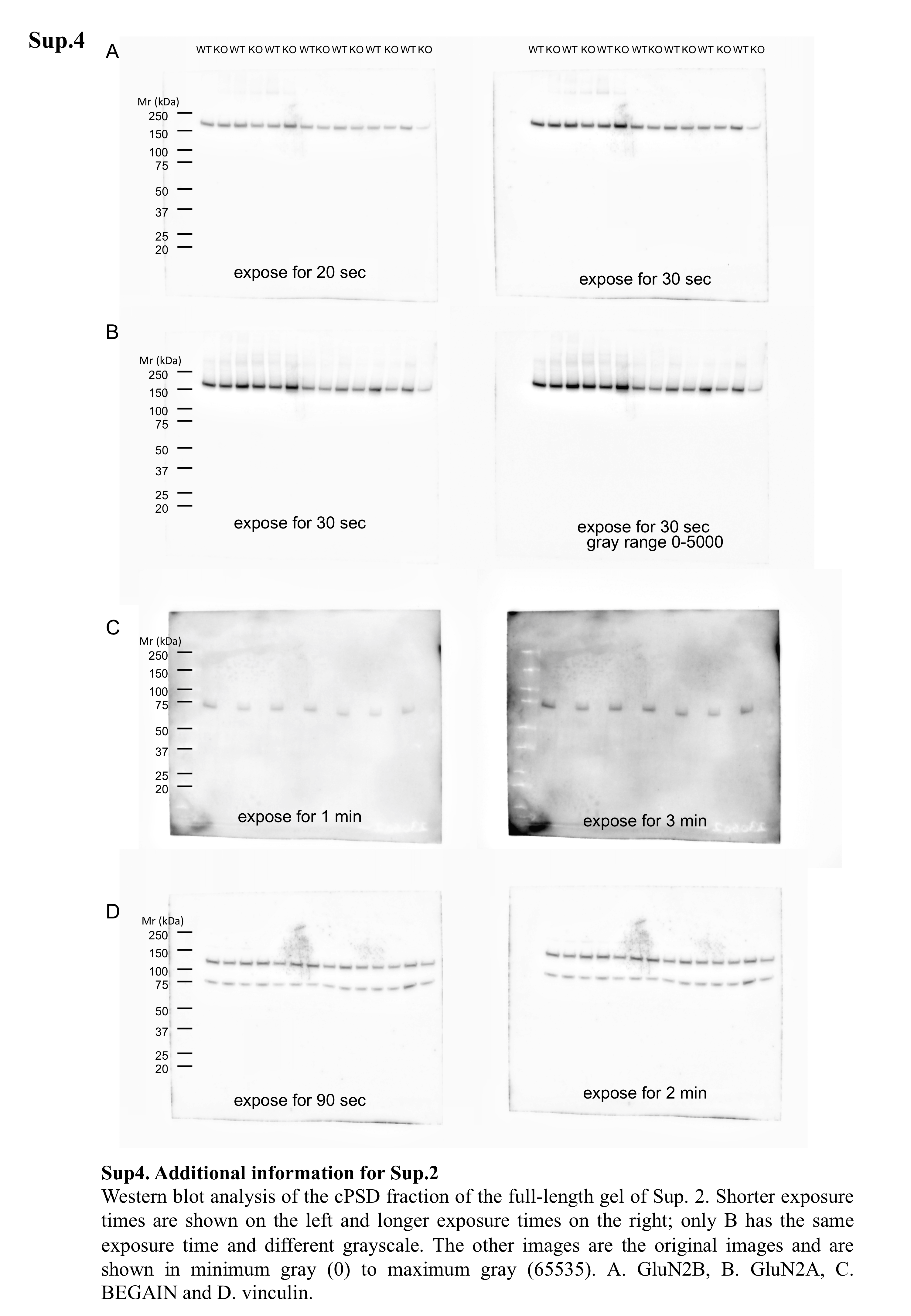

Supplement: Supplementary file 4 — Supplementary Information 4. [file 41598_2023_49537_MOESM4_ESM.tiff]
